# Case Report: Management of rectal squamous cell carcinoma - a treatment dilemma

**DOI:** 10.12688/f1000research.24033.1

**Published:** 2020-06-03

**Authors:** Nathaniel A. Parker, Yasmine Hussein Agha, Charles Scott Buess, Daniel Lalich, Jeremy M. Deutsch

**Affiliations:** 1 University of Kansas School of Medicine, 1010 N Kansas St, Wichita, KS, 67214, USA; 2 Wesley Medical Center, 550 N. Hillside St, Wichita, KS, 67214, USA; 3 Cancer Center of Kansas, 818 N. Emporia #403, Wichita, KS, 67230, USA

**Keywords:** Squamous cell carcinoma, Rectum, Chemoradiotherapy, Radiotherapy

## Abstract

Primary rectal squamous cell carcinoma is rare compared to adenocarcinoma, which is the predominant histologic type most commonly discovered at the time of colorectal carcinoma diagnosis. Due to the infrequent nature of this malignancy, data on tumor pathogenesis and risk factors remains sparse. Moreover, no standardized therapeutic regimen exists. This report describes a case of advanced rectal squamous cell carcinoma diagnosed in a 46-year-old female who initially presented with abdominal pain. Her clinical course was uncomplicated and she responded well to the selected therapy. Much work remains to be accomplished for patients with rectal squamous cell carcinoma.

## Introduction

Adenocarcinoma comprises the vast majority of rectal cancers ^1^. As a result, primary rectal squamous cell carcinoma (RSCC) is exceedingly rare, occurring in approximately 0.10–0.25 per 1000 colorectal cancers ^2, 3^. The etiology, pathogenesis, and risk factors are poorly defined, and no general consensus exists regarding the optimal treatment regimen due to the rarity of this cancer. Review of the literature encompasses mostly case series and retrospective studies. Nevertheless, evidence-based management is essential for those who are diagnosed. This report describes a rare case of primary RSCC.

## Case report

A 46-year-old Caucasian female administrative assistant, for whom the only pertinent past medical history was chronic tobacco smoking, presented at the emergency department with the chief complaint of generalized abdominal pain. Symptom onset began two months prior to her initial presentation and had been progressively worsening.

**Figure 1.**
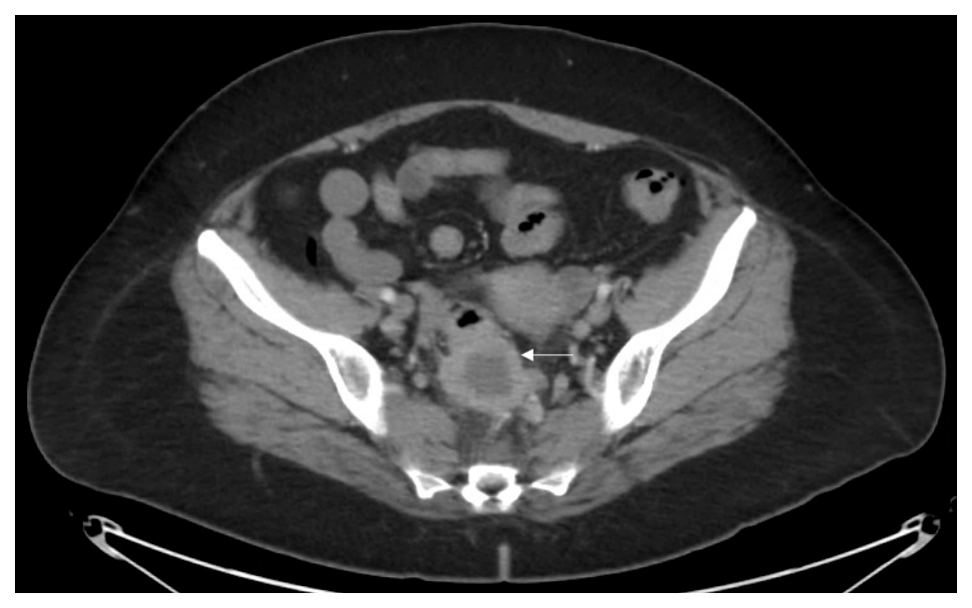
Abdominal imaging demonstrates a low-density mass involving the rectosigmoid colon. The rounded thick-walled structure measures approximately 4 cm ( *arrow*). There is some adjacent inflammation in the presacral space as well as prominent lymph nodes. Given the radiological findings the differential diagnosis includes transmural abscess versus inflammatory carcinoma of the sigmoid colon.

**Figure 2.**
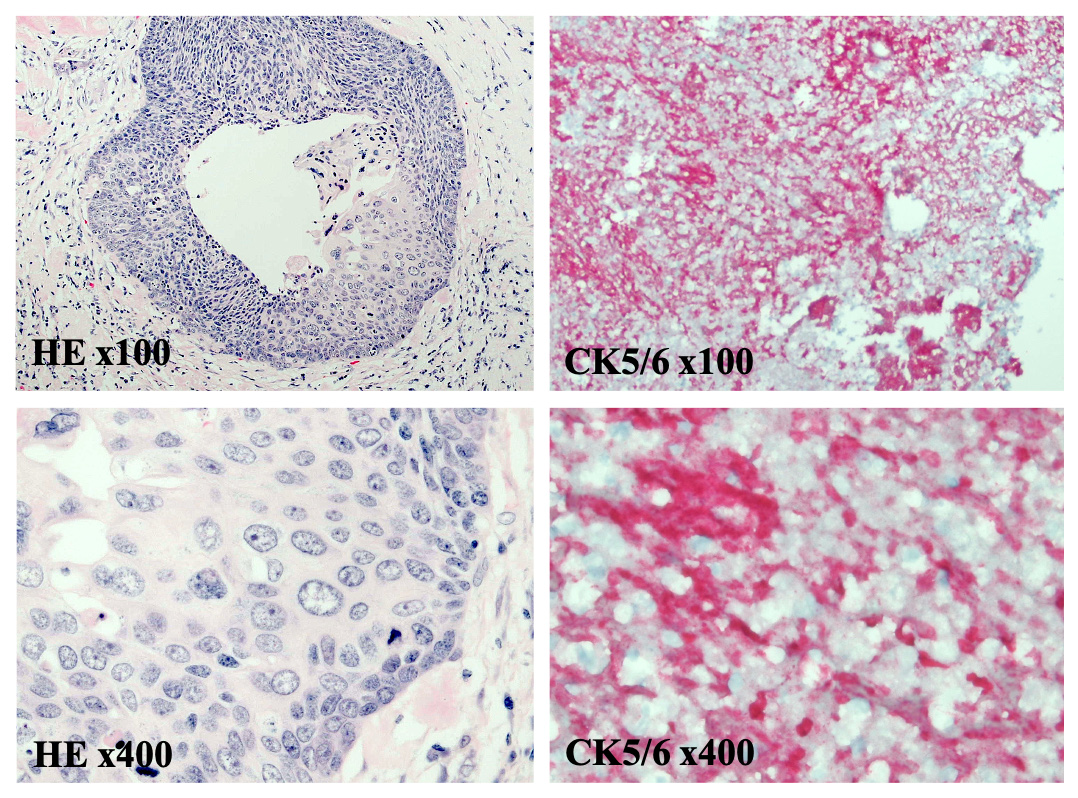
Pathology of the rectal mass demonstrates a squamous carcinoma. At medium and high power magnification, hematoxylin and eosin (HE) staining reveals sheets of poorly differentiated squamous cells invading the surrounding submucosal tissue (HE x40 and x100). Immunohistochemical staining for the squamous cell marker CK5/6, visualized by a cytoplasmic red-chromogen reaction, is positive (CK5/6 x40 and x100). Together histopathology and immunostaining show a poorly differentiated squamous cell carcinoma originating from rectal tissue.

**Figure 3.**
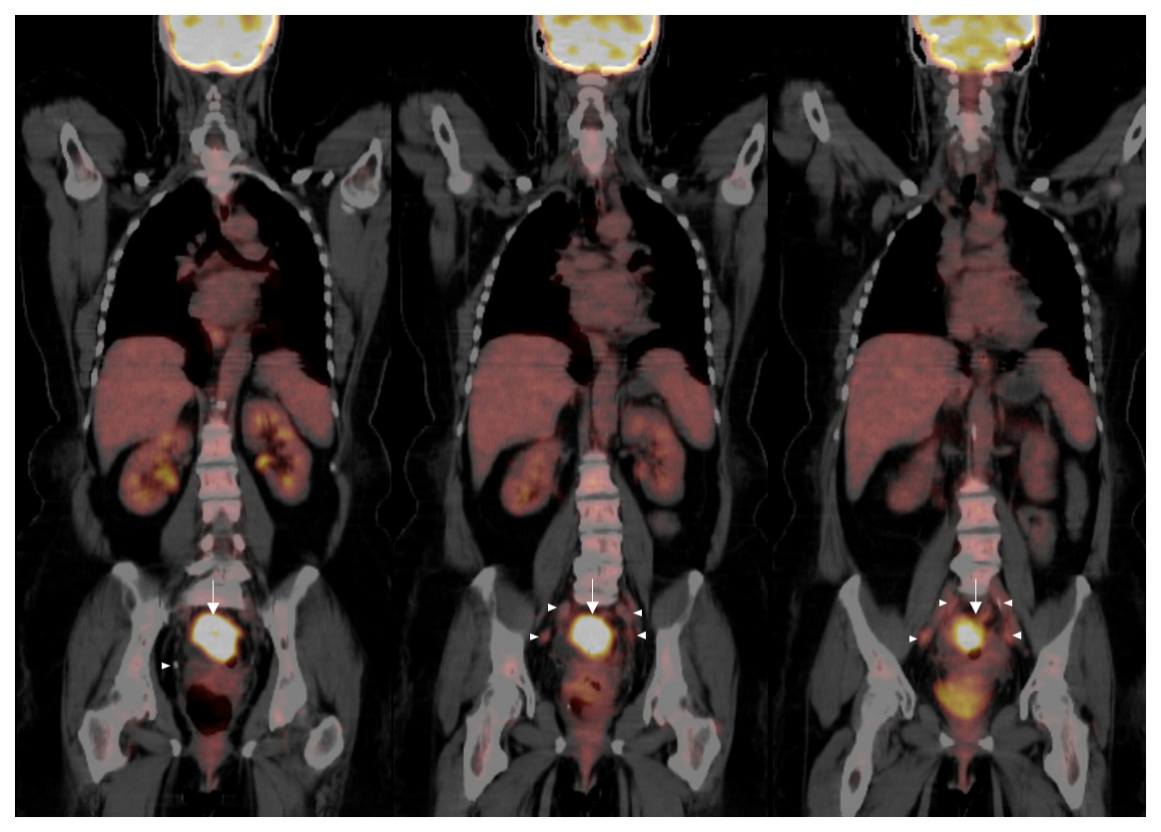
PET scans demonstrate a rectal malignancy. Imaging reveals a large focus of hypermetabolic activity in the rectosigmoid colon with a SUV of 16 and diffuse stranding in the region ( *arrows*). There are multiple slightly prominent perirectal lymph nodes with the maximal SUV of 3.3 ( *arrowheads*). There is presacral fat stranding and retroperitoneal lymphadenopathy, none of which exhibit hypermetabolism. No evidence of malignancy is noted above the diaphragm. Expected physiologic uptake of F-18 fluorodeoxyglucose is observed in the kidneys and brain. Given these findings, the rectal tumor was determined to be stage III rectal squamous cell carcinoma.

Vital signs and measurements were unremarkable. Physical examination was unremarkable. Serum laboratory evaluation was nonrevealing. Computerized tomography (CT) imaging of the abdomen and pelvis showed a sigmoid mass indicating a differential diagnosis of a transmural abscess versus a malignant inflammatory process in the sigmoid colon ( Figure 1). There was no evidence of distant metastatic disease. The patient underwent a diagnostic colonoscopy, which showed a rectosigmoid mass that was biopsied between 10 cm and 15 cm from the anal verge. Grossly, the mass was observed to have a flattened and friable mucosa. Histopathology favored a rare, poorly differentiated squamous cell carcinoma of the rectum. To confirm the impression of squamous differentiation, immunohistochemical (IHC) stains were performed on the biopsied specimens. The malignant cells showed strong cytokeratin 5/6 (CD5/6) immunoreactivity ( Figure 2). Thus, squamous cell carcinoma of the rectum was diagnosed. Due to the squamous cell origin of her rectal mass, she underwent subsequent gynecologic evaluation. Cervical and endometrial biopsies were negative for malignancy. For tumor staging and evaluate for distant metastatic disease, the patient had a positron emission tomography (PET) scan, which showed a rectosigmoid mass in the colon with a standardized uptake value (SUV) of 16 and multiple PET-avid pelvic lymph nodes with SUVs of 2–3 ( Figure 3).

**Figure 4.**
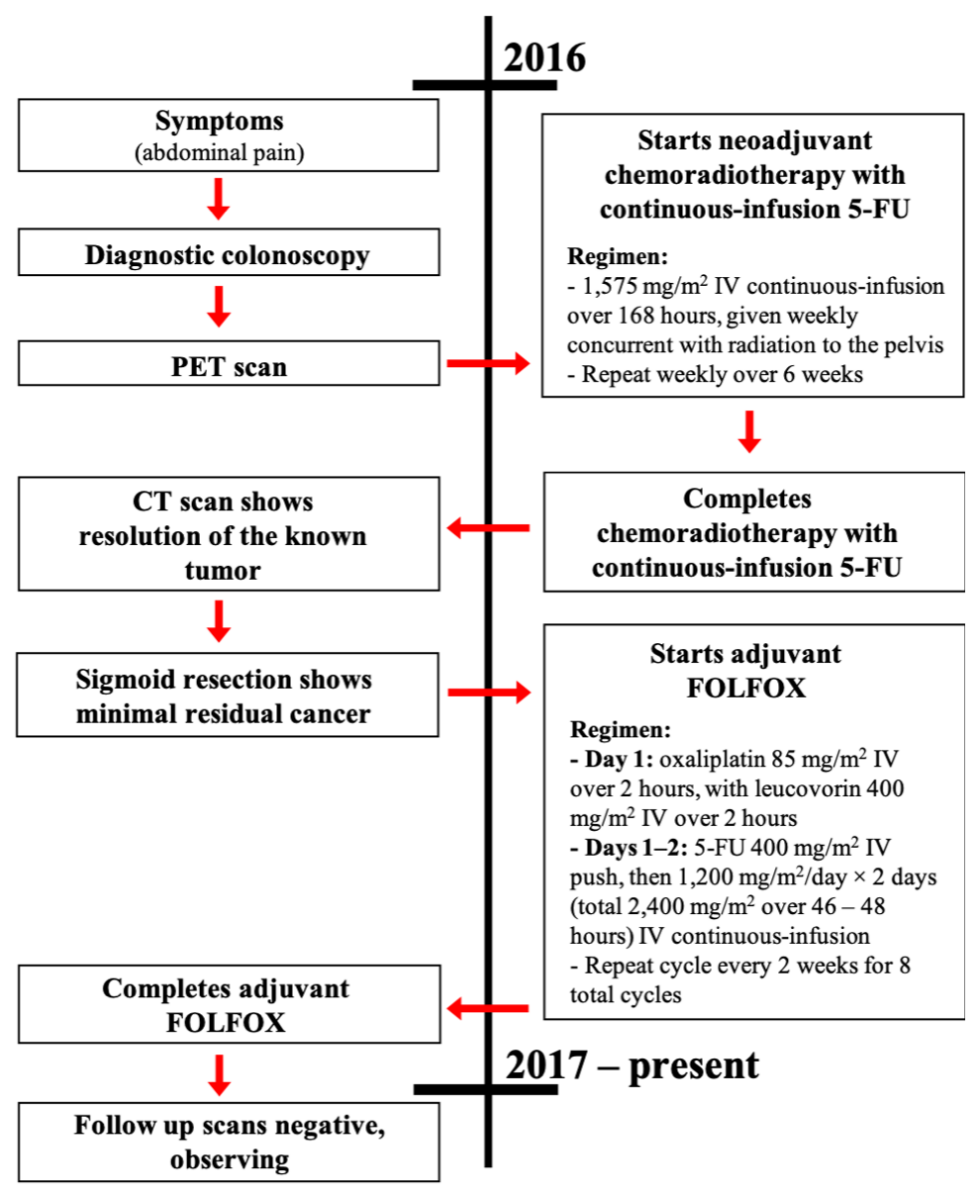
Case report timeline. Presented according to CARE guidelines.

Subsequently, she was diagnosed with stage III RSCC. Given the appearance of the tumor on CT scans, as well as the presence of PET-avid external iliac nodes in the perirectal region, neoadjuvant chemoradiation with radiation followed by surgical intervention was recommended. She was started on neoadjuvant chemotherapy with continuous-infusion 5-flurouracil (5-FU) with concomitant radiation. She received radiation therapy (28 treatments; total dose of 180 centiGrays) to her entire pelvis. Follow-up CT scans showed an excellent response and near resolution of the tumor. Subsequent PET scans displayed a low SUV in the primary tumor site with no additional uptake. She proceeded with sigmoid colon resection, with minimal residual carcinoma. Given the patient’s good response to chemotherapy and radiation, she was started on adjuvant chemotherapy with FOLFOX (leucovorin, 5-FU, and oxaliplatin) ( Figure 4).

Following adjuvant FOLFOX chemotherapy for six months, all of the patient’s consecutive surveillance CT scans have showed a complete resolution. This is consistent with a durable and long-lasting response to therapy for a rectal tumor that unusually originated from a poorly differentiated squamous cell carcinoma. The patient remains alive, healthy, and in complete remission following cessation of FOLFOX chemotherapy three years ago ( Figure 4).

## Discussion

Although RSCC has a similar presentation to rectal adenocarcinoma, its pathogenesis remains unclear and response to treatment is highly variable. Some of the most prominent risk factors include tobacco use, inflammatory bowel disease, radiotherapy and infections such as human immunodeficiency virus, human papilloma virus, amebiasis, and schistosomiasis ^4, 5^. Compared to adenocarcinoma of the rectum, RSCC occurs more often in younger Caucasian women with an average age of 60 years ^6, 7^. Patients clinically present with one or more of the following: gastrointestinal bleeding, changes in stool shape, diarrhea, constipation, tenesmus, weight loss and lower abdominal pain ^5^.

When histopathology is suggestive of RSCC, other more common etiologies such as anal squamous cell carcinoma, gynecological malignancy, and bowel fistula should be ruled out prior to establishing a definite diagnosis ^8^. Further evaluation and sampling can be achieved by colonoscopy and colposcopy. IHC plays an important role in differentiating RSCC from other histological subtypes. Although this specific IHC stain was not utilized in this case due to availability, cytokeratin CAM5.2, an epithelial marker, immunoreactivity suggests rectal tissue as the primary tumor site, rather than anal ^4^. Cytokeratin 7 and 20 stain glandular epithelia in the upper and lower gastrointestinal tract, respectively ^4, 9^. While these markers identify adenomatous malignancies, both are expected to be negative by IHC in tumors with a squamous cell origin.

Historically, radical surgery was recommended for RSCC. However, more recent analyses have shown improved outcomes following chemoradiation only in localized disease or preceding salvage surgical resection in advanced disease to reduce tumor burden ^2– 4, 6, 7, 10^. One of the main factors contributing to the discrepancy among the results and conclusions drawn is the lack of consistency in staging criteria used among all studies. This raises concern since management is based on tumor staging. Another factor that led to the paradigm shift was the amount of complications that arise following surgical intervention. Resection reduces the risk of death from the cancer itself. Patients often have worse outcomes and reduced overall survival due to the debilitating issues secondary to invasive interventions ^5^. Review of the literature reveals treatment choice can also be influenced by the perceived severity of the illness. As a result, patients with advanced disease and a poorer prognosis were often offered surgical resection rather than conservative management with chemotherapy. However, poor outcomes following surgical resection could have been attributed to complications rather than the extent of the disease itself. The current understanding is based on case series, and results are highly biased. This in turn raises the need for a standardized staging system. Furthermore, randomized controlled trials would help outline an effective management strategy based on disease severity.

It has been postulated that staging based on size rather depth of invasion is a better predictor of prognosis ^6^. Chemotherapeutic options for RSCC include 5-fluorouracil in combination with capecitabine or cisplatin. A five-year disease-free survival of 86% with chemoradiation only and 93% with chemoradiation plus salvage surgery has helped establish a benchmark for other therapeutic options ^11^. Four other case series involving patients with advanced RSCC have shown improved overall survival with chemoradiation as definitive management, as well as alternative salvage surgery ^2, 10, 12, 13^. However, these retrospective observations are derived from small cohort studies that reported multiple limitations. Thus, it would be difficult to determine if the findings can be generalized.

## Conclusion

This report presented a unique and rare case of a primary squamous cell carcinoma of the rectum. Most likely due to the extraordinarily low incidence of colorectal tumors having squamous cell origins, the etiology, pathogenesis, and risk factors for RSCC remain poorly understood. As a result, no standardized therapeutic regimen exists. Historically successful regimens for more common colorectal cancers, such as adenocarcinomas, will likely continue to be widely used in practice until additional therapeutic options are elucidated. Recently, overall survival has been shown to be improved for RSCC patients when certain regimens are used. However, this data comes from retrospective small cohort studies. Much work remains to be accomplished for patients with RSCC.

## Data availability

All data underlying the results are available as part of the article and no additional source data are required.

## Consent

Written informed consent for publication of their clinical details and clinical images was obtained from the patient.
