# Supplementary material for: Case Report: Management of rectal squamous cell carcinoma - a treatment dilemma
Source: F1000Res. 2020 Jun 3;9:503. [Version 1] doi: 10.12688/f1000research.24033.1 (PMC7445558; doi:10.12688/f1000research.24033.1)
Supplement: Peer review history

# Peer review: Reviewer response for version 1

**DOI:** 10.5256/f1000research.26511.r67425

**Published:** 2020-08-24

**Authors:** Mukesh S. Paudel

**Affiliations:** 1 Gastroenterology, Lumbini Medical College, Palpa, Nepal

The paper describes a rare case of squamous cell carcinoma of the rectum, which was diagnosed by colonoscopy and histopathological examination. The write-up is good and this paper will be a valueable addition to the current existing literature on this topic. The methodology of diagnosis and the treatment protocol have been well explained in the current paper.

Are enough details provided of any physical examination and diagnostic tests, treatment given and outcomes?

Yes

Is the case presented with sufficient detail to be useful for other practitioners?

Yes

Is sufficient discussion included of the importance of the findings and their relevance to future understanding of disease processes, diagnosis or treatment?

Yes

Is the background of the case’s history and progression described in sufficient detail?

Yes

Reviewer Expertise:

Colonoscopy, IBS, Liver disease

I confirm that I have read this submission and believe that I have an appropriate level of expertise to confirm that it is of an acceptable scientific standard.

# Peer review: Reviewer response for version 1

**DOI:** 10.5256/f1000research.26511.r65674

**Published:** 2020-06-30

**Authors:** George S. Stoyanov

**Affiliations:** 1 General and Clinical Pathology/Forensic Medicine and Deontology, Medical University of Varna, Varna, Bulgaria

- Based on the clinical description, the tumor seems to be in the sigmoid colon, which indeed is a rare localization. The authors however use the terms colon and rectum interchangeably. Rectal SCC carcinoma is common and the ethological factor there is most often HPV infection. 
- Regarding patient information - it is too much revealing to indicate the profession of the patient. Please replace with the statement that her field of work is not associated with this kind of malignancy. 
- On the diagnostic report, please specify TNM of the tumor and G as well. What did the gynecological report show, was there presence of HPV-associated changes in the cervical smear? That information could provide some information on the pathogenesis of the condition. 
- On the report of the operative materials, please provide the TRG pf the tumor.
- In the discussion, RSCC does not share many of the hallmarks of AC - no mucus and obstructions are much less common.
- General comment on writing style: in some places the text is written very confusingly e.g.: "Although this specific IHC stain was not utilized in this case due to availability, cytokeratin CAM5.2, an epithelial marker, immunoreactivity suggests rectal tissue as the primary tumor site, rather than anal". The discussion needs to focus more on the analysis of the patient and the data you have stated in the introduction such as "no general consensus exists regarding the optimal treatment regimen due to the rarity of this cancer". Histology is of sub-par quality - in the H&E stained slides I am convinced that that is SCC, but I am not convinced on the location of the tumor, please provide a new slide which is more informative. The quality of the IHC is very bad, there is no counter staining on the cellular nucleus and the pattern on reactivity is more suggestive of tumor necrosis rather than that of any SCC - these need to be addressed. The abstract is way too short and seems a little bit detached from the article main text - please restructure it so be more coherent with the data provided in the manuscript.

Are enough details provided of any physical examination and diagnostic tests, treatment given and outcomes?

Yes

Is the case presented with sufficient detail to be useful for other practitioners?

Partly

Is sufficient discussion included of the importance of the findings and their relevance to future understanding of disease processes, diagnosis or treatment?

No

Is the background of the case’s history and progression described in sufficient detail?

Partly

Reviewer Expertise:

pathology, immunohistochemistry, oncology

I confirm that I have read this submission and believe that I have an appropriate level of expertise to confirm that it is of an acceptable scientific standard, however I have significant reservations, as outlined above.

# Response

**Published:** 2020-07-20

**Authors:** Nathaniel Parker

**Affiliations:** Kansas University School of Medicine, USA

Based on the clinical description, the tumor seems to be in the sigmoid colon, which indeed is a rare localization. The authors however use the terms colon and rectum interchangeably. Rectal SCC carcinoma is common and the ethological factor there is most often HPV infection. 

*- This is to reflect the dynamic titles that were used in operative and pathology reports to describe this case (e.g. rectal vs. rectosigmoid). In terms of HPV, these details were not clinically significant to oncologist involved with the case, and thus were not reported.* 

Regarding patient information - it is too much revealing to indicate the profession of the patient. Please replace with the statement that her field of work is not associated with this kind of malignancy. 

*- This is a biased and non-constructive criticism However, if it satisfies the peer reviewer, this vocational information was made available at the request of the Editorial Board. Please ensure you are update on the peer review standards of F1000Research.*

On the diagnostic report, please specify TNM of the tumor and G as well. What did the gynecological report show, was there presence of HPV-associated changes in the cervical smear? That information could provide some information on the pathogenesis of the condition. 

*- All relevant information has been provided in the case.*

On the report of the operative materials, please provide the TRG pf the tumor.

*- No TRG information was available to report.*

In the discussion, RSCC does not share many of the hallmarks of AC - no mucus and obstructions are much less common.General comment on writing style: in some places the text is written very confusingly e.g.: "Although this specific IHC stain was not utilized in this case due to availability, cytokeratin CAM5.2, an epithelial marker, immunoreactivity suggests rectal tissue as the primary tumor site, rather than anal". The discussion needs to focus more on the analysis of the patient and the data you have stated in the introduction such as "no general consensus exists regarding the optimal treatment regimen due to the rarity of this cancer". Histology is of sub-par quality - in the H&E stained slides I am convinced that that is SCC, but I am not convinced on the location of the tumor, please provide a new slide which is more informative. The quality of the IHC is very bad, there is no counter staining on the cellular nucleus and the pattern on reactivity is more suggestive of tumor necrosis rather than that of any SCC - these need to be addressed. The abstract is way too short and seems a little bit detached from the article main text - please restructure it so be more coherent with the data provided in the manuscript.

*- These criticisms are biased and/or non-constructive.*

*- All available and relevant information has been reported in the case.*

# Peer review: Reviewer response for version 1

**DOI:** 10.5256/f1000research.26511.r64793

**Published:** 2020-06-29

**Authors:** Pratiksha Singh

**Affiliations:** 1 Mercer County Community College, Trenton, NJ, USA

The case report is well described in terms of investigations performed and the treatment modality selected. It helps the practitioners by adding to the limited number of case reports and presentations available. However, the authors fail to describe the patient's symptoms and physical examination in sufficient detail:

1. The chief complaint in an emergency department as abdominal pain requires a further detailed description of presenting symptoms especially pertaining to relevant negative history about bowel habit changes, bleeding per rectum and weight loss.
2. It would help to describe smoking as pack years rather than chronic tobacco smoking for better interpretation.
3. The authors mention measurements that were unremarkable along with vital signs, it would be helpful to describe what these were (Guerra, 2006 ^1^).

During the staging process, it would be helpful to know the relevant system used (AJCC 7th or 8th edition staging for rectal carcinoma (Tong *et al.*, 2018 ^2^).

 During discussion, the authors mention "Compared to adenocarcinoma of the rectum, RSCC occurs more often in younger Caucasian women with an average age of 60 years". A recent update mentions the age of the patients usually range from 39-93 years with average 63 years with no apparent ethnic predisposition (Guerra, 2006 ^1^).

 It would also help if in the discussion the authors could explain how this particular case was unique. 

 Thank you for giving the opportunity to review and learn from your case report.

Are enough details provided of any physical examination and diagnostic tests, treatment given and outcomes?

Partly

Is the case presented with sufficient detail to be useful for other practitioners?

Partly

Is sufficient discussion included of the importance of the findings and their relevance to future understanding of disease processes, diagnosis or treatment?

Yes

Is the background of the case’s history and progression described in sufficient detail?

No

Reviewer Expertise:

Limited by knowledge of immunohistochemistry.

I confirm that I have read this submission and believe that I have an appropriate level of expertise to confirm that it is of an acceptable scientific standard, however I have significant reservations, as outlined above.

## References

[1] : Primary squamous cell carcinoma of the rectum: An update and implications for treatment. *World J Gastrointest Surg*.2016;8(3) : 10.4240/wjgs.v8.i3.252 252-65 10.4240/wjgs.v8.i3.252 27022453PMC4807327

[2] : Comparison of the eighth version of the American Joint Committee on Cancer manual to the seventh version for colorectal cancer: A retrospective review of our data. *World J Clin Oncol*.2018;9(7) : 10.5306/wjco.v9.i7.148 148-161 10.5306/wjco.v9.i7.148 30425940PMC6230917

# Response

**Published:** 2020-07-20

**Authors:** Nathaniel Parker

**Affiliations:** Kansas University School of Medicine, USA

Thank you for the review. The authors responses are listed below peer review comments

The chief complaint in an emergency department as abdominal pain requires a further detailed description of presenting symptoms especially pertaining to relevant negative history about bowel habit changes, bleeding per rectum and weight loss.

*- All available details from the case presentation that were pertinent and/or positive have been listed.*

It would help to describe smoking as pack years rather than chronic tobacco smoking for better interpretation.

*- Smoking pack years was unknown to the authors.*

The authors mention measurements that were unremarkable along with vital signs, it would be helpful to describe what these were (Guerra, 2006 ^1^).

*- All available and relevant details from the case have been listed.*

During the staging process, it would be helpful to know the relevant system used (AJCC 7th or 8th edition staging for rectal carcinoma (Tong *et al.*, 2018 ^2^).

*- No formal staging process, such as the one mentioned, was not used during this case's work-up and management process. Thus, it would not be factual to present the case in such a manner.*

During discussion, the authors mention "Compared to adenocarcinoma of the rectum, RSCC occurs more often in younger Caucasian women with an average age of 60 years". A recent update mentions the age of the patients usually range from 39-93 years with average 63 years with no apparent ethnic predisposition (Guerra, 20061).

*- Please see reference numbers 6 and 7, which are more recent.*

It would also help if in the discussion the authors could explain how this particular case was unique. 

*- Please see the Abstract and Conclusion sections.*
